# A retrospective evaluation of the impact of patient ethnicity on the use of epidural analgesia or blood transfusions in children undergoing major oncologic surgery

**DOI:** 10.1186/s13741-019-0117-z

**Published:** 2019-06-20

**Authors:** Pascal Owusu-Agyemang, Juan P. Cata, Ravish Kapoor, Antoinette Van Meter, Acsa M. Zavala, Uduak U. Williams, January Y. Tsai, Lei Feng, Andrea Hayes-Jordan

**Affiliations:** 1 Department of Anesthesiology and Perioperative Medicine, The University of Texas MD Anderson Cancer Center, 1515 Holcombe Boulevard, Unit 0409, Houston, USA; 2 Anesthesiology and Surgical Oncology Research Group, Houston, USA; 3 Department of Biostatistics, The University of Texas MD Anderson Cancer Center, Houston, USA; 4 Division of Pediatric Surgery, University of North Carolina at Chapel Hill School of Medicine, Chapel Hill, USA

**Keywords:** Ethnicity, Anesthesia, Children, Transfusion, Epidural, Pain

## Abstract

**Background:**

The impact of patient ethnicity on healthcare delivery is well documented. In this study of children who had undergone open abdominal or pelvic surgery for tumor resection, we sought to compare the use of epidural analgesia or intraoperative blood transfusions between Caucasian and non-Caucasian children.

**Methods:**

A retrospective study of 139 children was performed. Logistic regression models were used to evaluate the association between the specified perioperative factors and patient ethnicity.

**Results:**

The average age (standard deviation) was 11 years (± 5), 50% were female, and 58% were Caucasian. Compared to Caucasian children, non-Caucasian children were younger (difference in mean, − 2.6 years; 95% confidence interval [− 4.3, − 0.9], *p* = 0.003), underwent shorter procedures (difference in mean anesthesia minutes, − 134; 95% confidence interval [−  230, − 39], *p* = 0.006), and had a lower proportion of patients who received epidural analgesia (66% versus 81%, *p* = 0.042) or blood transfusions (48% versus 65%, *p* = 0.039). In the adjusted model, patient ethnicity was not associated with the receipt of epidural analgesia (odds ratio 0.53, 95% confidence interval [0.23, 1.21], *p* = 0.132) or blood transfusions (odds ratio 0.77, 95% confidence interval [0.29, 2.04], *p* = 0.600). The use of epidural analgesia or blood transfusions was associated with abnormal coagulation factors (odds ratio 0.32, 95% confidence interval [0.14, 0.71], *p* = 0.005) and the duration of surgery (odds ratio 1.007, 95% confidence interval [1.005, 1.009], *p* < 0.001), respectively.

**Conclusion:**

In this study of children who had undergone major oncologic surgery, the use of epidural analgesia or blood transfusions was not associated with patient ethnicity.

## Background

The impact of patient ethnicity on healthcare delivery has been documented in several developed countries (Egede, 2006; Salway et al., 2016; Brzoska, 2018) and across different subspecialties of medicine (Clegg et al., 2002; Haider et al., 2013). In anesthesia, compared to Caucasian patients, African American and Hispanic patients have been shown to be more averse to the acceptance of epidural analgesia (Ochroch et al., 2007; Orejuela et al., 2012). Black race has also been associated with higher rates of blood transfusion during certain surgical procedures (Maher et al., 2018; Qian et al., 2014).

The reasons for ethnic-based differences in the delivery of healthcare are manifold and include factors such as socioeconomic status (Ochroch et al., 2007), differences in access to healthcare (Chen et al., 2016), and differences in stages of disease at presentation (Austin et al., 2015). The extent to which ethnic-based differences exist in the use of epidural analgesia in children undergoing major oncologic surgery is unclear. Likewise, there is limited data on ethnic-based differences in blood transfusion requirements in children undergoing major oncologic surgery.

Identification of ethnic-based differences in care delivery within a practice setting could potentially provide an opportunity for quality improvement. Based on this premise, we conducted a retrospective study of children and adolescents who had undergone open abdominal or pelvic tumor resection by a single surgeon. Our primary objective was to determine whether there were any ethnic-based differences in the use of epidural analgesia or in intraoperative blood transfusions between Caucasian and non-Caucasian children. Our secondary objective was to compare the early postoperative outcomes of non-Caucasian children to those of Caucasian children. Based on the results of previous studies (Ochroch et al., 2007; Orejuela et al., 2012; Maher et al., 2018; Qian et al., 2014), we hypothesized that compared to Caucasian children, non-Caucasian children would have a lesser likelihood of receiving epidural analgesia and have higher rates of blood transfusion.

## Methods

This single institution retrospective study was approved by the Institutional Review Board of the University of Texas, MD Anderson Cancer Center (IRB # PA 16-0160). A waiver of informed consent was granted.

### Data collection

**Fig. 1:**
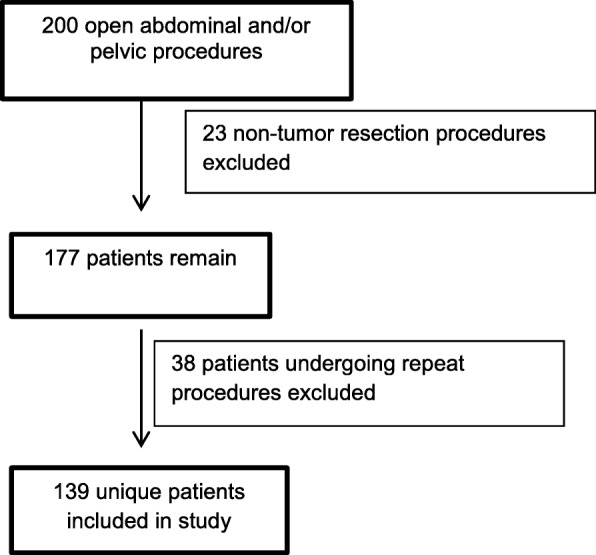
Consort diagram of the chart selection process

Medical records of patients ≤ 19 years of age who had undergone open abdominal or pelvic surgery between January 2006 and January 2017 were reviewed. Patients who had undergone procedures that did not involve tumor resection and those who had undergone repeat or subsequent procedures were excluded. A consort diagram of the chart selection process is presented as Fig. 1.

After a unique group of patients had been identified, baseline and demographic data including age, gender, American Society of Anesthesiologists physical status score, body mass index, ethnicity, preoperative opioid use, and the type of surgical procedure were recorded. Preoperative laboratory values including the hemoglobin level, platelet count, prothrombin time, partial thromboplastin time, and serum creatinine and albumin levels were included. Recorded intraoperative variables included the use of epidural analgesia (Yes/No), estimated blood loss, crystalloid, colloid and blood transfusions, intraoperative opioid administration, and anesthesia duration. Average pain scores over the first 24 h after surgery, the first postoperative hemoglobin value, length of stay, the incidence of high-grade complications (≥ Clavien-Dindo grade 3) (Clavien et al., 2009), and readmissions within 30 days after surgery were also recorded. Perioperative characteristics of non-Caucasian (Black, Hispanic, Asian, and Arabic) children were compared to those of Caucasian (White) children.

### Statistical analysis

Patient demographics, diagnoses, and laboratory measurements were summarized through descriptive statistics. Wilcoxon rank sum and Kruskal-Wallis tests were used to compare continuous distributions across ethnic groups. Fisher’s exact or chi-square test was used to evaluate the association between patient characteristics and ethnicity. Logistic regression models were fitted to estimate the effect of ethnicity on anesthesia delivery. Statistical software SAS 9.4 (SAS, Cary, NC) was used for all the analyses. A *p* value of ≤ 0.05 was considered statistically significant.

## Results

A total of 139 children and adolescents were included in the study. Of these, 80 (58%) were Caucasian, and 59 (42%) were non-Caucasian (3% Arabic, 4% Asian, 16% Black or African American, and 19% Hispanic). The average age (standard deviation) was 11 years (± 5), and 50% were female. Perioperative characteristics of the entire study population are presented in Table 1.

**Table 1:** Perioperative characteristics of Caucasian and non-Caucasian children who had undergone abdominal/pelvic tumor resection

Variable (mean ± standard deviation)	Non-Caucasian (*n* = 59)	Caucasian (*n* = 80)	Difference in mean (95% CI)	*p* value	Entire study population (*n* = 139)
Age, years	9.7 ± 5.0	12.3 ± 5.0	− 2.6 (− 4.3, − 0.9)	0.003	11.2 ± 5.1
Gender, *n* (%)				0.659	
Female	28 (47)	41 (51)			69 (50)
Male	31 (53)	39 (49)			70 (50)
ASA, *n* (%)				0.085	
3 or 4	46 (78)	71 (89)			117 (84)
Body mass index	19 ± 4	20 ± 6	− 1 (− 3, 0)	0.083	20 ± 5
Preoperative opioid use, *n* (%)	7 (12)	26 (33)		0.005	33 (24)
Baseline laboratory values					
Hemoglobin, mg/dL	11.0 ± 2.6	11.1 ± 1.7	− 0.1 (− 0.8, 0.7)	0.880	11.1 ± 2.1
Albumin, mg/dL	4.1 ± 0.6	4.1 ± 0.4	0 (− 0.2, 0.2)	0.907	4.1 ± 0.5
Creatinine, mg/dL	0.5 ± 0.2	0.6 ± 0.4	− 0.1 (− 0.2, 0.0)	0.108	0.6 ± 0.3
Platelet count, K/μL	255 ± 144	235 ± 143	20 (− 29, 69)	0.424	244 ± 143
PT, s	14.1 ± 1.4	13.9 ± 1.1	0.2 (− 0.3, 0.7)	0.397	14.0 ± 1.2
PTT, s	36.9 ± 12.5	34.4 ± 11.7	2.5 (− 1.9, 6.9)	0.251	35.4 ± 12.1
Abnormal coagulation or platelets, *n* (%)	25 (42)	25 (31)		0.177	50 (36)
Surgical procedures, *n* (%)					
Abdominal	10 (17)	9 (11)		0.023	19 (14)
Pelvic	8 (14)	4 (5)			12 (9)
Abdomino-pelvic	29 (49)	59 (74)			88 (63)
Retroperitoneal	12 (20)	8 (10)			20 (14)
Anesthesia duration, min	552 ± 282	686 ± 278	− 134 (− 230, − 39)	0.006	629 ± 289
Epidural analgesia, *n* (%)	39 (66)	65 (81)		0.042	104 (75)
Intraoperative opioids, MED	2.1 ± 2.1	2.1 ± 1.8	0 (− 0.7, 0.7)	0.768	2.1 ± 1.9
Estimated blood loss, ml/kg	13 ± 19	16 ± 26	− 3 (− 11, 4)	0.401	15 ± 24
Transfusions					
Crystalloid, ml/kg	54 ± 41	67 ± 40	− 13 (− 27, 1)	0.059	61 ± 41
Colloid, ml/kg	20 ± 21	29 ± 22	− 9 (− 16, − 2)	0.013	25 ± 22
Blood/blood products, *n* (%)	28 (48)	52 (65)		0.039	80 (58)
Postop hemoglobin, mg/dL	11.2 ± 1.8	11.3 ± 1.7	− 0.1 (− 0.7, 0.5)	0.720	11.2 ± 1.8
Pain score postop day 1	1.6 ± 1.7	1.9 ± 1.8	− 0.3 (− 0.9, 0.3)	0.398	1.8 ± 1.8
High-grade complications, *n* (%)	10 (17)	10 (13)		0.460	20 (14)
Length of stay, days	12.6 ± 14.5	12.3 ± 7.1	0.3 (− 3.8, 4.4)	0.878	12 ± 11
S30-day readmissions, *n* (%)	8 (14)	14 (18)		0.529	22 (16)

*T* test was used for the continuous variables. Fisher’s exact test or chi-square test was used for the categorical variables. *n* (%) number of patients (percentage of a subgroup), *ASA* American Society of Anesthesiologists physical status score, *PT* prothrombin time, *PTT* partial thromboplastin time, *MED* morphine dose equivalents

### Baseline characteristics of Caucasian and non-Caucasian patients

Compared to Caucasian children, non-Caucasian children were younger (difference in mean − 2.6 years, 95% confidence interval [− 4.3, − 0.9] and were less likely to be on prescription opioids preoperatively (12% versus 33%, *p* = 0.005). Other differences in baseline characteristics between the two study groups were not statistically significant (Table 1).

### Intraoperative care

Compared to Caucasian children, a lower percentage of non-Caucasian children received epidural analgesia for surgery (66% versus 81%, *p* = 0.042). Non-Caucasian children also underwent shorter procedures (difference in mean anesthesia minutes, − 134, 95% confidence interval [− 230, − 39], *p* = 0.006), received a lesser volume crystalloid (difference in mean − 13 ml/kg, 95% confidence interval [− 27, 1], *p* = 0.059), and colloid (difference in mean − 9 ml/kg, 95% confidence interval [− 16, − 2], *p* = 0.013), and had a lower percentage of patients who received blood transfusions (48% versus 65%, *p* = 0.039). There were no other statistically significant differences in intraoperative care (Table 1).

### Differences in the use of epidural analgesia

In the univariate analysis, non-Caucasian children were less likely to receive epidural analgesia (odds ratio 0.45, 95% confidence interval [0.21, 0.98], *p* = 0.044). After adjusting for preoperative opioid use and abnormal preoperative coagulation parameters, the association between patient ethnicity and the receipt of epidural analgesia was not statistically significant (odds ratio 0.53, 95% confidence interval [0.23, 1.21], *p* = 0.132). In the adjusted model, the odds of receiving epidural analgesia were significantly lower in children with abnormal coagulation parameters (odds ratio 0.32, 95% confidence interval [0.14, 0.71], *p* = 0.005), Table 2.

**Table 2:** Odds ratio estimates on the use of epidural analgesia

Preoperative variable	Univariate OR (95% CI) *p* value	Multivariate OR (95% CI) *p* value
Preoperative opioid use	1.72 (0.64, 4.59) 0.281	1.47 (0.52, 4.19) 0.472
Abnormal coagulation or platelets	0.29 (0.13, 0.65) 0.003	0.32 (0.14, 0.71) 0.005
Non-Caucasian	0.45 (0.21, 0.98) 0.044	0.53 (0.23, 1.21) 0.132

### Differences in the use of blood transfusions

In the univariate analysis, non-Caucasian children were less likely to receive a blood transfusion (odds ratio 0.49, 95% confidence interval [0.25, 0.97], *p* = 0.040). After adjusting for American Society of Anesthesiologists physical status score, body mass index, preoperative opioid use, and anesthesia duration, the association between ethnicity and blood transfusion was not statistically significant (odds ratio 0.77, 95% confidence interval [0.29, 2.04], *p* = 0.60). In the adjusted model, the transfusion of blood was associated with the duration of anesthesia (odds ratio 1.007, 95% confidence interval [1.005, 1.009], *p* < 0.001), Table 3.

**Table 3:** Odds ratio estimates for the intraoperative transfusion of blood

Perioperative variable	Univariate OR (95% CI) *p* value	Multivariate OR (95% CI) *p* value
ASA 3 or 4	3.56 (1.35, 9.40) 0.011	1.56 (0.41, 5.94) 0.514
BMI ≥ 25	1.84 (0.68, 4.98) 0.233	1.91 (0.44. 8.18) 0.386
Preoperative opioid use	1.00 (0.45, 2.21) 0.998	0.58 (0.19, 1.76) 0.335
Anesthesia duration	1.007 (1.005, 1.009) < 0.0001	1.007 (1.005, 1.009) < 0.001
Non-Caucasian	0.49 (0.25, 0.97) 0.040	0.77 (0.29, 2.04) 0.600

*ASA* American Society of Anesthesiologists physical status score, *BMI* body mass index

### Postoperative course

Postoperative variables including the first postoperative hemoglobin value, average pain scores over the first 24 h after surgery, the incidence of high-grade complications, length of stay, and 30-day readmissions were similar between the two study groups (Table 1).

## Discussion

In this retrospective study of children and adolescents who had undergone major abdominal or pelvic surgery for tumor resection, the use of epidural analgesia or blood transfusions was not associated with patient ethnicity. Furthermore, early postoperative outcomes including pain scores over the first 24 h after surgery, the first postoperative hemoglobin value, the incidence of high-grade complications, length of stay, and 30-day readmission rates were similar between Caucasian and non-Caucasian children.

The influence of patient ethnicity on the acceptance of epidural analgesia has been reported in several studies (Ochroch et al., 2007; Orejuela et al., 2012). For example, in a scripted telephone survey of patients who were scheduled for elective surgery, African American race was the only factor that predicted refusal or acceptance of epidural analgesia (Ochroch et al., 2007). In another prospective observational study of labor pain management in a predominantly Hispanic population, 72% of the patients declined epidural upon admission (Orejuela et al., 2012). The reasons for non-acceptance of epidural analgesia in minority populations remain unclear. Factors such as socioeconomic status and level of education have been suggested (Ochroch et al., 2007). However, in a study assessing ethnic disparities in the provision of epidural analgesia to low-income patients, rates were lower for Black, Hispanic, and Asian women, than for White women. These differences in epidural use persisted even after controlling for age, rural-urban residence, and availability of anesthesiologists, suggesting the role of cultural preferences in the observed disparity (Rust et al., 2004).

At a large academic institution such as ours, where all patients possess health insurance, patient preference may play a more significant role in the decision to use epidural analgesia than socioeconomic factors. In our practice, education about the benefits of epidural analgesia is provided during both the surgical and anesthesia preoperative visits and perhaps results in an improved informed decision-making process. Furthermore, although not widely reported, studies suggest patients’ choices about anesthetic options are influenced by discussions with their surgeons. For example, in the scripted telephone survey by Ochroch and colleagues (Ochroch et al., 2007), the acceptance rate for epidural analgesia increased from 64% when it was recommended by only the anesthesiologist to 78.5% when it was recommended by both the surgeon and anesthesiologist. Based on the above, we are led to speculate that our practice of educating patients during both the surgical and anesthesia preoperative visits may have influenced parents’ perceptions about epidural analgesia and reduced the number of non-Caucasian parents who may have declined to consent to the use of epidural analgesia for their children. On the other hand, not all studies have demonstrated an association between patient ethnicity and the use of neuraxial analgesia. For example, in a retrospective propensity-matched study of adults undergoing knee and hip replacement surgery, ethnic-based differences in the use of neuraxial analgesia were not observed after controlling for several important demographic and clinical factors (Elsharydah et al., 2018). This raises the possibility that in patient groups with relatively similar preoperative characteristics, such as ours, the association between the use of epidural analgesia and patient ethnicity may be less significant.

The results of studies evaluating ethnic disparities in pain management have been conflicting (Goyal et al., 2015; Nafiu et al., 2017). For example, in a cross-sectional study of children diagnosed with appendicitis, Black children were undertreated for both moderate and severe pain (Goyal et al., 2015). The authors suggested the existence of a different threshold for the treatment of pain in Black children. To the contrary, in a prospective observational study of children who underwent outpatient surgery, the children of ethnic minorities were more likely to receive intravenous opioids for the management of mild pain (Nafiu et al., 2017). In our study, the doses of opioids administered during surgery and pain scores over the first 24 h after surgery were similar between Caucasian and non-Caucasian children. Our findings seem to add to the conflicting nature of publications on this topic. This may be related to the complex interaction several factors including the source of pain (Goyal et al., 2015), child and parental attitudes to pain (Twycross et al., 2015), ethnic disparities in pain perception (Green et al., 2003), and language barriers (Fortier et al., 2011). The intraoperative and early postoperative periods are also unique, in that, factors such as hemodynamic variables and the type and duration of surgery may have a more significant impact on the treatment of pain, than patient ethnicity, or parental influence. Although not entirely clear, the difference in findings might also suggest that protocoled care, as utilized in the immediate postoperative period and in the aforementioned prospective observational study, may more closely address patients’ needs.

Studies suggest that compared to Caucasian patients, Black patients undergoing major surgery may have higher rates of blood transfusion (Maher et al., 2018; Qian et al., 2014). In our study, blood transfusion rates were lower in non-Caucasian children. However, the adjusted model did not demonstrate an association between blood transfusion and patient ethnicity but, as expected, demonstrated a significant association with the duration of surgery. Grouping of Black patients with other non-Caucasian patients may have influenced our results. However, similar blood transfusion rates have been reported between Black and White patients undergoing colectomy (Qian et al., 2014). This raises the possibility that ethnic disparities in blood transfusion may be dependent on the type of surgery or diagnosis.

An important finding of this study was that early postoperative outcomes including the first postoperative hemoglobin value, the incidence of high-grade complications, length of stay, and 30-day readmission rates were similar between Caucasian and non-Caucasian children. Our findings may be a reflection of our institutional practice of continuously reviewing patient outcomes and instituting measures towards improvement. Furthermore, several of the children in this study were treated according to established multidisciplinary study protocols, some of which contained specific preoperative, intraoperative, and postoperative instructions (Subbiah et al., 2018). For example, in children undergoing cytoreductive surgery with hyperthermic intraperitoneal chemotherapy, strict perioperative blood and fluid transfusion goals were set. This was initiated preoperatively by admitting children the day before surgery and initiating a crystalloid infusion at 1.5 times the maintenance rate during fasting and bowel preparation. Intraoperatively, crystalloids and/or colloids were transfused to maintain a urine output ≥ 2 ml/kg/h (Owusu-Agyemang et al., 2012). Over the first 48 postoperative hours, crystalloid solutions were transfused at 1.5 times the maintenance rate. In addition, fluid losses (urine output and output from abdominal drains) were replaced on a 1:1 ratio with crystalloid solutions (Mauricio et al., 2010). With regard to the transfusion of blood or blood products, a hemoglobin value of less than 10 g/dL, or an abnormal coagulation value, triggered a conversation with the surgeon about the appropriateness of transfusion. This protocoled approach may have reduced potential differences in care and resulted in similar outcomes.

Another significant finding of this study was that Caucasian children underwent longer procedures, had a higher proportion of procedures that involved tumor resection from both the abdomen and pelvis, had higher rates of blood transfusion, and received higher volumes of crystalloid and colloid. This suggests Caucasian children may have presented with a disease of a more widespread or advanced nature than their non-Caucasian counterparts. Other studies in children with cancer have shown the contrary, with Black and Hispanic children more likely to present with advanced stages of solid tumor malignancies than White children (Baker et al., 2002; Henderson et al., 2011). The reasons for the difference in our findings are not entirely clear and deserve further investigation. One possibility could be that more Caucasian children with advanced disease were referred to our institution for treatment. Despite the availability of health insurance, socioeconomic factors may have limited the ability of some families of lower socioeconomic status to travel and relocate for the duration of treatment. Studies have shown that socioeconomic status is strongly associated with ethnicity, with Blacks and Hispanics usually demonstrating lower median incomes than Whites (Williams et al., 2016).

Our study has several limitations that are mostly related to its retrospective nature and small sample size. Therefore, significant selection and information biases are an important weakness of this work. Furthermore, we did not have access to information about socioeconomic status and other factors that could influence patients’ or caregivers’ preferences on analgesia. Additionally, grouping together of all Caucasian and non-Caucasian children may have limited our ability to determine specific issues associated with specific ethnicities. With regard to epidural analgesia, limiting the sample size to one surgeon decreased variability in the information that was presented to families during the surgical preoperative visit. This may have been a source of bias in this study.

## Conclusions

In this study of children who had undergone major oncologic surgery by a single surgeon, differences in the use of epidural analgesia or blood transfusions were associated with perioperative factors, rather than patient ethnicity. Further large-scale research is required to determine whether ethic-based disparities exist in the use of epidural analgesia or blood transfusions in children undergoing major surgery.

## Data Availability

The data that support the findings of this study are available from The University of Texas MD Anderson Cancer Center, but restrictions apply to the availability of these data, which were used under license for the current study, and so are not publicly available. Data are however available from the authors upon reasonable request and with permission of The University of Texas MD Anderson Cancer Center.
